# Toxic Effects of Acute Water Selenium Exposure on *Litopenaeus vannamei*: Survival, Physiological Responses, Transcriptome, and Intestinal Microbiota

**DOI:** 10.3390/ani15121792

**Published:** 2025-06-18

**Authors:** Xinghui Luo, Jian Chen, Asare Derrick, Gongyu Li, Hongming Wang, Zhihao Xue, Lili Shi, Shuang Zhang

**Affiliations:** 1 College of Fisheries, Guangdong Ocean University, Zhanjiang 524088, China; daluo0528@163.com (X.L.); chenjian@catas.cn (J.C.); nasare8713@gmail.com (A.D.); sligongyu200007@163.com (G.L.); wanghongming97@163.com (H.W.); xzh102904@163.com (Z.X.); shill@gdou.edu.cn (L.S.); 2 Zhanjiang Experimental Station, Chinese Academy of Tropical Agricultural Sciences, Zhanjiang 524013, China; 3 Shenzhen Institute of Guangdong Ocean University, Shenzhen 518120, China

**Keywords:** selenium, acute exposure, antioxidant, intestinal microbiota, transcriptome

## Abstract

Excess selenium (Se) can cause a variety of toxic effects in aquatic animals. However, there is currently a lack of comprehensive studies about the toxicity effects of Se in culture water on shrimp. Based on the 96 h acute toxicity test, which confirmed the 96 h LC_50_ of Se (Na_2_SeO_3_) for *Litopenaeus vannamei* as 2.69 mg/L, *L. vannamei* (7.25 ± 0.05 g) were divided into three groups (named CON, S1 and S2) and exposed to Se at concentrations of 0, 0.0269 (0.01 × 96 h LC_50_), and 0.269 (0.1 × 96 h LC_50_) mg/L in the water for 72 h, respectively. The toxic effects of Se exposure on *L. vannamei* were evaluated based on histopathology, oxidative stress, immunity, apoptosis, transcriptional responses, and intestinal microbiota. Results demonstrated that Se exposure induced structural damage to the hepatopancreas of *L. vannamei*, including hepatocyte vacuolation and necrosis. Compared to the CON group, serum Caspase-3 activity significantly increased, while Bcl-2 activity markedly decreased in the S1 and S2 groups (*p* < 0.05). No significant differences in Bax activity were observed among groups (*p* > 0.05). ROS content, as well as activities of SOD, PO, GSH-PX, LYS, AKP, and ACP, exhibited an upward trend under Se exposure (*p* < 0.05). However, MDA levels showed no significant intergroup differences (*p* > 0.05). Hemocyte transcriptomic analysis revealed 2103 differentially expressed genes (DEGs) (1294 upregulated, 809 downregulated) in the S2 group compared to CON. GO enrichment indicated significant enrichment of DEGs in cellular processes, binding, and cell components. KEGG pathway analysis highlighted prominent enrichment in ribosome biogenesis in eukaryotes, lysosome, cell cycle, and pancreatic secretion pathways. Intestinal microbiota analysis showed that the Shannon, Simpson, and Pielou indices in the S2 group were significantly lower than those in the CON group (*p* < 0.05). The relative abundance of *Vibrio* and *Acinetobacter* increased significantly in the S2 group, while *Enterococcus* and *Pseudomonas* decreased markedly (*p* < 0.05). In conclusion, Se exposure triggered elevated immune enzyme activities, induced oxidative damage and apoptosis, transcriptional level metabolic disorders, and disrupted intestinal microbiota structure in *L. vannamei*.

## Simple Summary

Selenium (Se) is an essential trace element, but excessive amounts can be toxic to aquatic animals. This study investigated how different concentrations of Se in water affect the Pacific white shrimp (*Litopenaeus vannamei*). The results showed that Se exposure caused damage to the shrimp’s hepatopancreas, increased the activity of immune and antioxidant enzymes, and led to cell death. In addition, the balance of gut bacteria was disrupted, with harmful bacteria increasing and beneficial bacteria decreasing. These findings suggest that too much Se in aquaculture water can harm shrimp health by affecting their internal organs, immune system, and gut microbiota.

## 1. Introduction

In recent years, the accelerated development of coastal regions has exacerbated marine environmental degradation. Among various environmental concerns, toxic contamination of aquatic ecosystems has attracted considerable global attention [1]. Unlike organic pollutants, heavy metals cannot be metabolized into less harmful substances within the organisms themselves, although some external biological processes may help mitigate their toxicity [2]. Even worse, they tend to bioaccumulate through the food chain, creating persistent environmental hazards. The aquaculture industry is particularly vulnerable to heavy metal contamination, as these toxic elements can infiltrate breeding systems through contaminated feed, aquaculture equipment, and water sources, ultimately inducing a range of deleterious effects, including reproductive impairments, morphological abnormalities, and elevated mortality [3,4]. Additionally, the bioaccumulation of heavy metals in edible aquatic organisms poses substantial threats to human health, with excessive dietary intake causing a variety of toxicological effects, including cancer, neurasthenia, leukemia, and neurological disorders [5]. For these reasons, elucidating the toxicological mechanisms of heavy metals and their effects on aquatic organisms has become crucial.

Selenium (Se), a metalloid compound with properties between metals and non-metals, was first discovered by Swedish chemist Jons Jacob Berzelius in 1818 [6]. Recognized for its beneficial effects in maintaining antioxidant defenses, repairing DNA damage, and protecting the liver, Se is commonly supplemented in the feed to boost productivity and improve disease resistance in aquatic animals [7]. However, the margin between its nutritional requirement and toxicity in aquatic organisms is remarkably narrow, making excessive addition potentially harmful. Research has indicated that long-term dietary Se exposure induces oxidative stress in the zebrafish brain, subsequently altering the integrity of the dopamine system, including disturbances in DNA synthesis, release, uptake, and receptor activation [8]. In Atlantic salmon (*Salmo salar*), dietary supplementation with 15 mg/kg sodium selenite significantly suppressed the feeding activity and growth performance while reducing circulating levels of free fatty acids, monoacylglycerol, diacylglycerol, and endocannabinoids, which suggested Se-induced dysregulation of lipid homeostasis through interference with key metabolic pathways [9]. Aquatic species exhibit heightened sensitivity to selenosis [10]. Generally, Se concentrations in natural waters are relatively low. However, with the increase in human activities such as power generation, oil refining, and mining, elevated levels of Se (primarily in its inorganic forms) are becoming more common, which poses significant risks to aquatic ecosystems. Therefore, it is essential to evaluate the safe concentration of Se in aquaculture water.

Crustaceans play a vital role in maintaining ecological stability and are very sensitive to environmental pollution, such as plastics, pesticides, and heavy metals, which can be affected even in small concentrations [11]. As a crustacean with a high aquaculture yield worldwide, *Litopenaeus vannamei* is sensitive to changes in various physicochemical properties in the water environment and is often used as a common model organism to study the toxic effects of environmental pollutants, including heavy metals [12,13]. Cd^2+^ exposure in *L. vannamei* resulted in an increase in reactive oxygen species, a decrease in total blood count (THC), and a sharp decrease in both apoptosis rates and expression of immune-related genes [14]. Similarly, Pb exposure in *L. vannamei* increased intestinal levels of O^2-^, H_2_O_2_, and MDA, decreased the abundance of Proteobacteria and Firmicutes, and disrupted microbial diversity [15]. In general, the toxic effects of exposure to several heavy metals on *L. vannamei* have been investigated, except for Se.

In fact, there are only a few studies about the toxic effects of water Se on aquatic animals, which focused on zebrafish (*Danio rerio*), with limited studies addressing other aquatic species. In *D. rerio,* Se exposure induces Se accumulation in the body, impairing embryonic and tissue development, altering cell apoptosis, and triggering oxidative stress [16]. Given the dual role of Se as both an essential trace element and a potential environmental poison at high concentrations, the toxicity of water Se to *L. vannamei* was studied in this study, from the aspects of histopathology, oxidative stress, immunity, apoptosis, transcriptional responses, and intestinal microbiota. The findings could provide valuable insights into the mechanisms underlying Se-induced toxicity, contributing to a deeper understanding of aquatic health risks, which could offer a theoretical foundation for improving health management strategies and promoting sustainable aquaculture practices.

## 2. Materials and Method

### 2.1. Experimental Animal Management

*L. vannamei* (7.25 ± 0.05 g) were obtained from Guangdong Yuehai Feed Group Co., Ltd., and acclimatized for a week in outdoor concrete tanks (4.5 m × 4.9 m × 1.8 m) at the Donghai Island aquaculture base. Water parameters were maintained: temperature 20.0–27.0 °C, salinity 26–30 g/L, pH 7.7–8.0, dissolved oxygen ≥6.0 mg/L, and ammonia <0.05 mg/L. During acclimation, shrimp were fed commercial feed at a daily ration equivalent to 5% of their body weight, administered three times daily (08:00, 13:00, and 19:00).

### 2.2. Acute Toxicity Assay

A 96 h acute toxicity test was conducted following the Guidelines for Acute Toxicity Testing in Aquatic Organisms (EPA/ROC, 1998) using a static renewal method. Sodium selenite (Na_2_SeO_3_) was added to the water at nominal concentrations of 0.1, 0.5, 2.5, 12.5, 62.5, and 125 mg/L. A total of 540 shrimp were divided into 6 groups and reared in the water with different Na_2_SeO_3_ concentrations, respectively. Each group had three replicates, with 30 shrimp per replicate. Test solutions in each tank were renewed daily to maintain consistent Se concentrations, while water parameters matched those described in Section 2.2. Shrimp were fasted during the test, and mortality was recorded at 24 h, 48 h, 72 h, and 96 h. Mortality of shrimp was defined by the absence of response to sterile forceps contact, and deceased individuals were promptly removed. The 96 h median lethal concentration (LC_50_) and its 95% confidence interval were calculated using Karber’s method [17]. According to the results of the 96 h LC_50_ determination, 360 shrimp were divided into three groups (CON, S1, and S2) and exposed to low-dose selenium treatments corresponding to 0, 1/100, and 1/10 of the LC_50_ value. Each group included three replicates with 40 shrimp per tank (0.3 m^3^). Water parameters, consistent with Section 2.1, were monitored and recorded daily.

### 2.3. Sample Collection and Processing

Upon experimental termination, the hepatopancreas of 3 shrimp, hemolymph of 6 shrimp, and intestines of 3 shrimp in a tank were collected after ice anesthesia. The same tissue types from 3 shrimp were pooled to form one biological replicate, and three biological replicates were collected per group. The hepatopancreas sample was excised and preserved in Bouin’s fixative (Phygene, Fuzhou, China) for histopathological analysis. Hemolymph was aspirated from the ventral sinus of shrimp using a 1 mL sterile syringe and pooled into two centrifuge tubes. One hemolymph sample was stored at 4 °C for 12 h and then was centrifuged (3000× *g*, 10 min, 4 °C) to collect supernatants for the detection of the activities of superoxide dismutase (SOD), phenol oxidase (PO), catalase (CAT), glutathione peroxidase (GSH-Px), lysozyme (LYS), acid phosphatase (ACP), alkaline phosphatase (AKP), and Caspase 3, and the contents of reactive oxygen species (ROS), malondialdehyde (MDA), B-cell lymphoma-2 (Bcl-2) protein, and Bcl2-associated X (Bax) protein. Another hemolymph sample was centrifuged (3000× *g*, 10 min, 4 °C) immediately after collection, and the hemocytes were collected. The hemocytes and intestine samples were immediately flash-frozen in liquid nitrogen and subsequently stored at −80 °C for long-term preservation, for use in transcriptome analysis and microbiota analysis, respectively.

### 2.4. Histological Analysis

Hepatopancreas tissue samples were fixed in Bouin’s fixative for 24 h and subsequently dehydrated. The dehydrated tissues were then stored in 75% ethanol before being sent to Seville Company for sectioning and staining (5 μm thickness). Following hematoxylin–eosin (HE) staining, the sections were examined and imaged under a Nikon microscope (Nikon, Minato-ku, Japan) equipped with a Mshot digital imaging system. Tissue integrity was preserved throughout processing to ensure high-quality sections.

### 2.5. Biochemical Analysis

Using the method of double-antibody one-step sandwich enzyme-linked immunosorbent assay (ELISA), the activities of SOD, PO, CAT, GSH-Px, LYS, ACP, AKP, and Caspase 3, and the contents of ROS, MDA, Bcl-2 protein, and Bax protein were detected by ELISA Kits ML305751, ML923919, ML454948, ML113425, ML695266, ML036384, ML036449, ML543724, ML955621, ML555268, ML552691, and ML931205, respectively. All the ELISA Kits were purchased from Shanghai Enzyme Link Biotechnology Co., Ltd. (Shanghai, China).

### 2.6. Transcriptome Analyze

Total RNA was extracted from hemocyte samples of L. vannamei across experimental groups (CON and S2) using TRIzol reagent (Invitrogen, Waltham, MA, USA). Genomic DNA was removed with DNase I (Takara, Shiga, Japan), and RNA quality was assessed for purity (NanoDrop 2000, Thermo Fisher Scientific, Waltham, MA, USA), integrity (Agilent 2100 Bioanalyzer, Agilent, Santa Clara, CA, USA), and RNA integrity number. Qualified RNA samples were submitted to Guangzhou Genedenovo Biotechnology Co., Ltd. (Guangzhou, China) for library preparation and paired-end sequencing (Illumina Hiseq2500 PE250, Illumina, Santa Clara, CA, USA).

Raw sequencing reads were processed using FASTP [18] to remove adapter sequences, low-quality reads (Q-score < 20), and reads with >10% ambiguous bases (N). Clean reads were aligned to the *L. vannamei* reference genome (NCBI Genome ID: 10710) using HISAT2 [19]. Transcript assembly and quantification were performed with StringTie [20], and gene expression levels were normalized to FPKM (fragments per kilobase per million mapped reads). Differentially expressed genes (DEGs) between CON and S2 groups were identified using DESeq2 [21], with thresholds of false discovery rate (FDR) < 0.05 and |log_2_(fold change) | ≥ 1. Functional annotation and enrichment analysis of DEGs were conducted via GO (Gene Ontology) and KEGG (Kyoto Encyclopedia of Genes and Genomes) databases. Raw transcriptome data were deposited in the NCBI Sequence Read Archive (Accession: PRJNA1241152).

### 2.7. qRT-PCR Verification of DEGs

To validate the transcriptome analysis, ten DEGs (five up-regulated, five down-regulated) were randomly selected. Total RNA from hemocyte samples was reverse-transcribed into cDNA using the Evo M-MLV RT Kit (Accurate Biotechnology, Hunan, China). Primers for target genes (Table 1) were designed with Primer Premier 5.0, and elongation factor 1α (EF1α: XM027373349) served as the reference gene. qPCR was performed on a LightCycler 480 II system (Roche, Basel, Switzerland) under the following conditions: 95 °C for 3 min, followed by 40 cycles of 95 °C for 10 s, 58 °C for 30 s, and 72 °C for 30 s. Relative gene expression was calculated using the 2^−ΔΔCt^ method, and the correlation between RNA-seq and qPCR results was assessed via linear regression analysis.

### 2.8. Intestinal Microbiota

Intestinal total bacterial DNA was extracted using the HiPure Soil DNA Kit (Magen, Guangzhou, China) according to the manufacturer’s instructions. The quality of the extracted DNA was assessed using a NanoDrop ND-2000 spectrophotometer (Thermo Fisher Scientific, USA). DNA samples meeting quality standards were subsequently used for amplification and sequencing. The full-length 16S rRNA gene was amplified using universal primers 27F (CCTACGGGNGGCWGCAG) and 1492R (GGACTACHVGGGTATCTAAT). After purification and quantification of the amplified products, libraries were constructed and subjected to long-read sequencing on the PacBio platform. Sequencing and bioinformatics analysis of the intestinal microbiota were conducted by Guangzhou Genedenovo Biotechnology Co., Ltd. (Guangzhou, China). The sequencing data have been deposited in the NCBI GenBank database under accession number PRJNA1236889.

### 2.9. Statistical Analyses

Experimental data were analyzed using one-way analysis of variance (ANOVA) and Tukey’s multiple comparisons, and graphical representations were generated using GraphPad Prism 8.0. Probit analysis was used to establish the LC_50_ value of Se. All quantitative results are expressed as mean ± standard deviation (SD). Statistical significance for all comparisons was defined as *p* < 0.05.

## 3. Results

### 3.1. Se Toxicity Thresholds

Table 2 shows cumulative mortality rates at 24 h intervals for *L. vannamei* exposed to various Se concentrations, revealing a clear dose–response relationship. No mortality occurred at 0.5 mg/L, whereas mortality reached 5% at 2.5 mg/L and 100% at 62.5 mg/L within 24 h. LC_50_ values decreased with exposure time, from 7.12 mg/L at 24 h to 3.74 mg/L at 48 h, and 2.69 mg/L at both 72 h and 96 h. Using the safety concentration formula (SC = 0.1 × 96 h LC_50_) yielded a safety concentration of 0.269 mg/L.

### 3.2. Hepatopancreatic Histopathology

**Figure 1:**
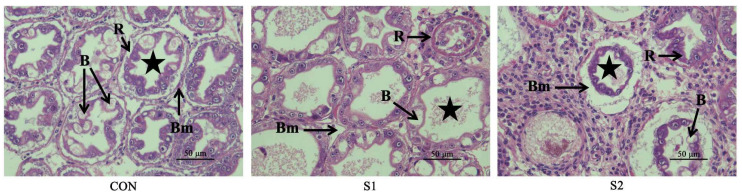
Effects of different Se concentrations on hepatopancreatic structure in *L. vannamei.* B: secretory cells B cells; R: adipocytes R cells; BM: cell membrane; star symbol: official; black bold arrows: lesions.

Histological examination of the hepatopancreas (Figure 1) revealed normal morphology in the CON group, with well-organized lumens and intact cellular architecture. In the S1 group, pathological changes included B-cell hypertrophy, partial luminal degeneration, basement membrane contraction, and nuclear enlargement. The S2 group exhibited more severe degeneration, including disrupted cell membranes, cytoplasmic leakage, loss of intracellular organization, and nuclear fragmentation. Overall, these lesions progressed in a dose-dependent manner.

### 3.3. Apoptotic Response

**Figure 2:**
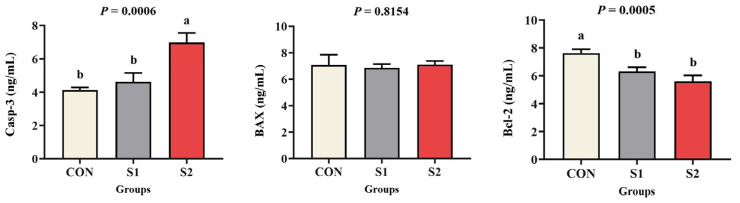
Effects of acute Se exposure in water on the levels of apoptotic enzymes in *L. vannamei*. The standard error ± mean (n = 3) was expressed as the difference between groups, different letters indicate significant differences exist among treatments (*p* < 0.05).

Figure 2 shows that caspase-3 activity was significantly higher in the S2 group than in the CON and S1 groups (*p* < 0.05). No significant difference in caspase-3 activity was detected between the cCON and S1 groups (*p* > 0.05). BAX activity did not differ significantly among any of the groups (*p* > 0.05). In contrast, Bcl-2 activity was significantly lower in both the S1 and S2 groups relative to the CON group (*p* < 0.05).

### 3.4. Oxidative Stress and Antioxidant Capacity

**Figure 3:**
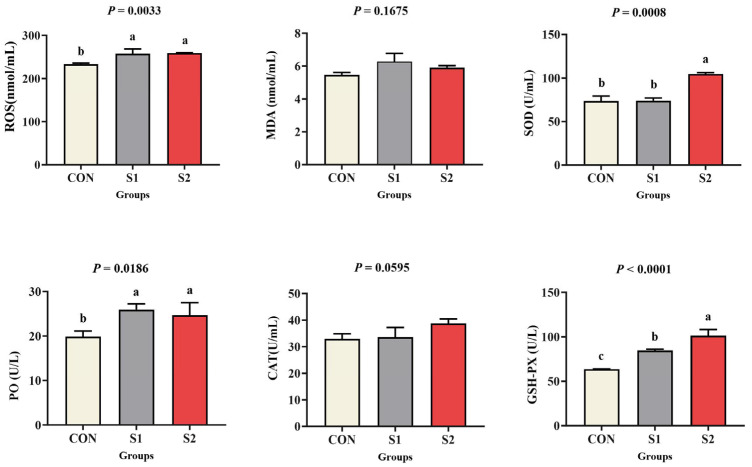
Effects of acute Se exposure on antioxidant enzymes and antioxidant capacity in *L. vannamei.* The standard error ± mean (n = 3) was expressed as the difference between groups, different letters indicate significant differences exist among treatments (*p* < 0.05).

Figure 3 shows that ROS and PO levels increased significantly in both Se-exposed groups compared to the CON group (*p* < 0.05). Activities of the antioxidant enzymes SOD and GSH-PX were also significantly elevated in the S1 and S2 groups compared with the CON group (*p* < 0.05). In contrast, MDA content and CAT activity remained unchanged across all groups (*p* > 0.05).

### 3.5. Immune Response

**Figure 4:**
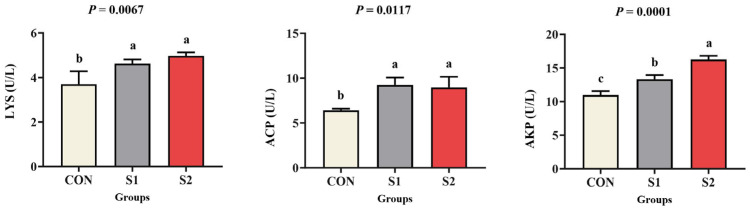
Effects of acute Se exposure on immunoenzyme activities in *L. vannamei*. The standard error ± mean (n = 3) was expressed as the difference between groups, different letters indicate significant differences exist among treatments (*p* < 0.05).

Figure 4 demonstrates the dose-responsive alterations in immune-related enzymatic activities under Se exposure. Compared to the CON group, both S1 and S2 groups exhibited significantly elevated LYS and ACP activities (*p* < 0.05). AKP activity displayed a progressive dose-dependent increase, with the highest activity observed in the S2 group (*p* < 0.05).

### 3.6. Transcriptome Analysis

#### 3.6.1. Sequencing Data Evaluation Statistics

To further elucidate the mechanisms underlying Se-induced hemocyte damage in *L. vannamei*, transcriptomic analysis was performed on hemocyte samples from the CON and S2 groups. As summarized in Table 3, an average of 6.67 billion and 6.88 billion raw reads were obtained from the CON and S2 groups, respectively. After quality filtering, 6.58 billion and 6.80 billion clean reads remained. The Q20 scores exceeded 96%, and Q30 scores were above 92% across all samples, with GC contents ranging from 45.90% to 47.67%, indicating high sequencing accuracy and reliability.

#### 3.6.2. Differential Gene Expression

**Figure 5:**
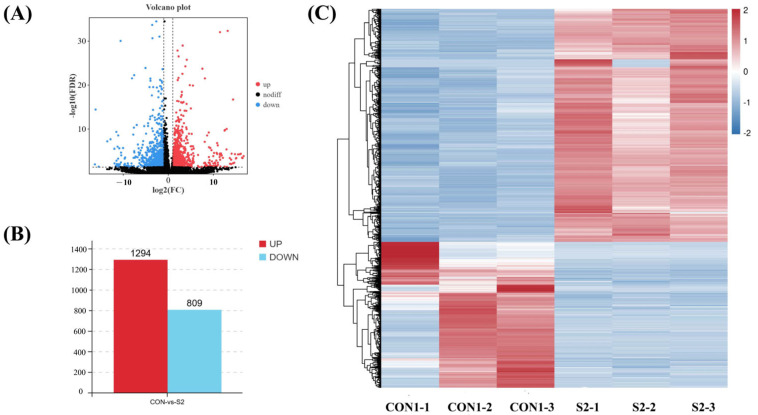
DEGs in hemocyte transcriptome of *L. vannamei* under Se stress. (**A**) Volcano diagram of DEGs; (**B**) histogram of DEGs; (**C**) heatmap distribution of DEGs.

Differential gene expression analysis identified a total of 2103 DEGs between the CON and S2 groups, comprising 1294 upregulated and 809 downregulated genes (Figure 5A,B). Hierarchical clustering analysis showed a clear separation between the two groups, and the heatmap visualization further illustrated distinct gene expression patterns (Figure 5C), suggesting a consistent transcriptional response to Se exposure.

#### 3.6.3. GO and KEGG Enrichment Analysis of DEGs

**Figure 6:**
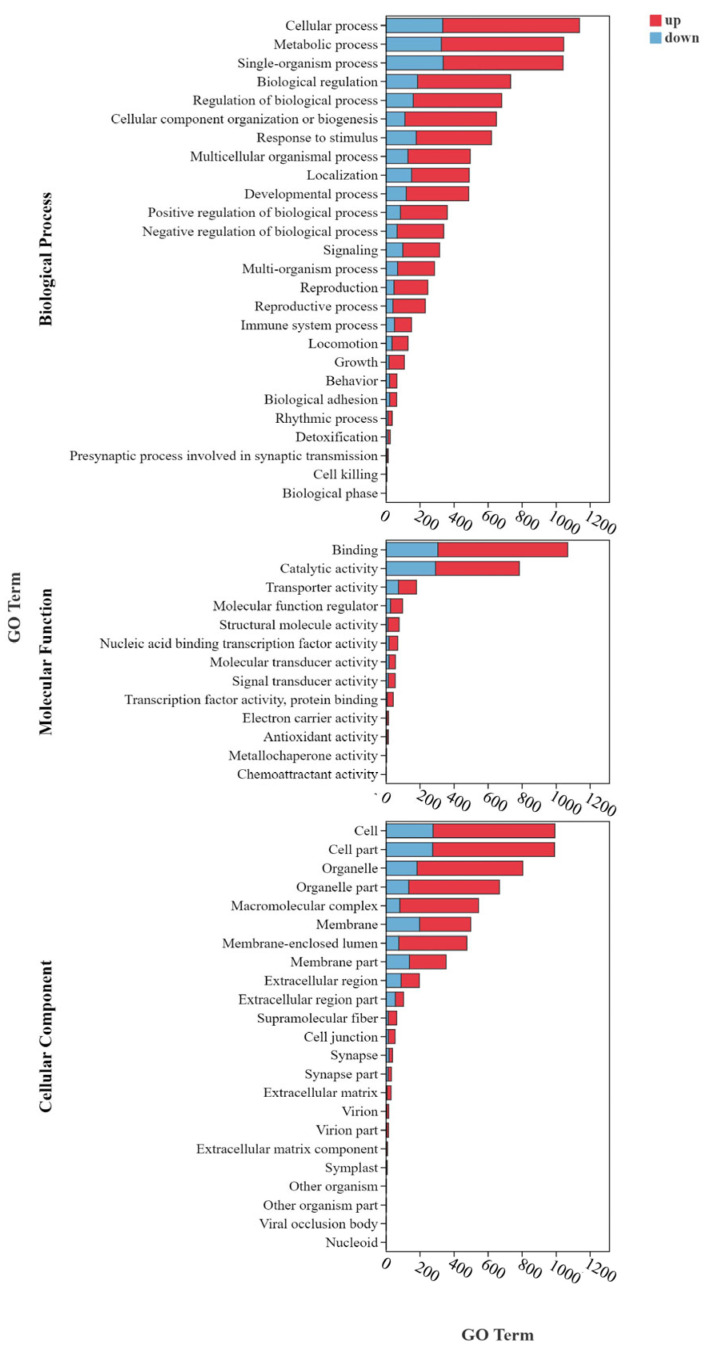
GO enrichment analysis of DEGs.

Functional annotation of DEGs was conducted using the GO and KEGG databases to elucidate the biological processes and pathways affected by Se exposure. GO enrichment analysis identified 66 significantly enriched terms across three major categories: biological process (BP), cellular component (CC), and molecular function (MF) (Figure 6). Within the MF category, DEGs were primarily enriched in transcription factor activity, protein binding, catalytic activity, signal transducer activity, transporter activity, electron carrier activity, and antioxidant activity. In the CC category, enrichment was observed in terms such as macromolecular complexes, extracellular region, membrane, nucleoid, cell junction, and extracellular matrix. For the BP category, DEGs were mainly associated with metabolic processes, cellular processes, immune system processes, biological adhesion, and reproductive and developmental processes. Additionally, genes involved in carboxylic acid, organic acid, and α-amino acid metabolism, as well as oxalate degradation and biosynthesis of organic acids, were significantly enriched, indicating that Se exposure triggers broad metabolic adjustments. 

**Figure 7:**
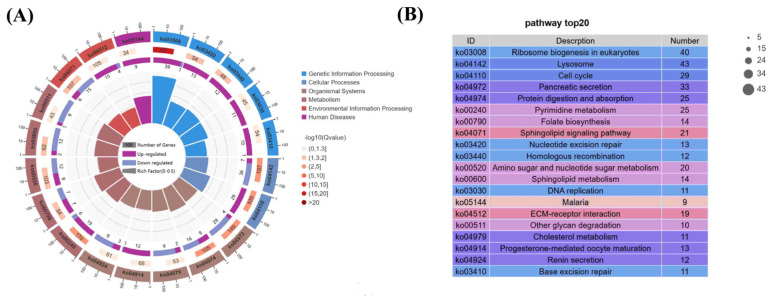
KEGG enrichment analysis of DEGs. (**A**) Enrichment circle plot; (**B**) Top 20 significantly enriched DEG pathways.

KEGG pathway enrichment analysis further revealed key molecular pathways associated with Se-induced hepatotoxicity. DEGs were mapped to six major functional domains, including metabolism, cellular processes, environmental and genetic information processing, biological systems, and human diseases (Figure 7A). Notably, significant enrichment was observed in pathways related to carbohydrate metabolism, digestive function, infectious disease, and immune responses. Among the top 20 significantly enriched pathways (Figure 7B), lysosome, ribosome biogenesis in eukaryotes, pancreatic secretion, cell cycle, and pyrimidine metabolism were particularly prominent. Ribosome biogenesis, in particular, exhibited strong enrichment and clustering, suggesting its critical involvement in the molecular response to Se-induced hemocyte damage. Detailed pathway information is provided in Table 4.

### 3.7. qPCR Validation of RNA-Seq Results

**Figure 8:**
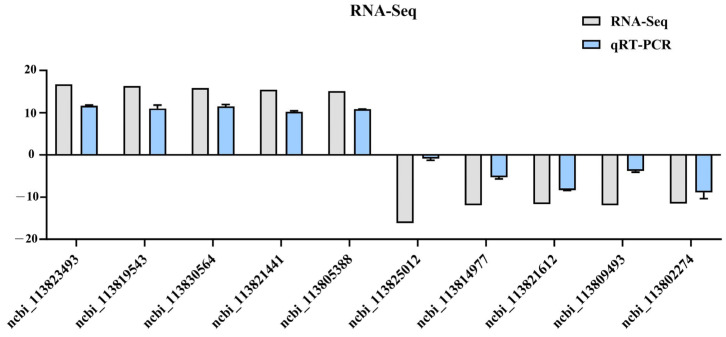
Validation of DEGs by qPCR.

To validate the RNA-seq findings, a total of 10 DEGs exhibiting significant expression differences between CON and S2 groups were subjected to RT-qPCR analysis. As illustrated in Figure 8, the expression profiles of these DEGs demonstrated complete concordance with the transcriptomic data, confirming the reliability of the sequencing results. 

### 3.8. Intestinal Microbiota Analysis

#### 3.8.1. Diversity of Intestinal Microbiota

**Figure 9:**
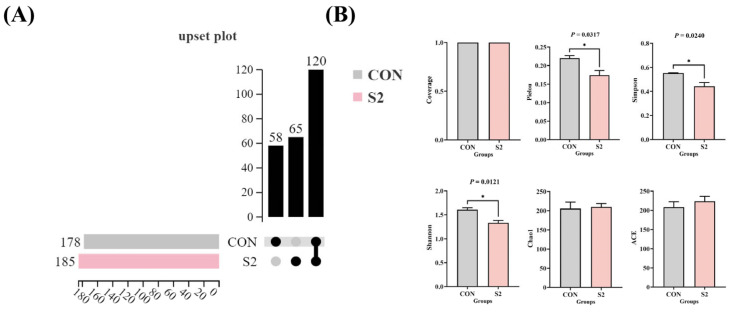
Changes of acute Se exposure in water on the intestinal microbial diversity of *L. vannamei*. (**A**) UpSet plot; (**B**) α diversity index. * indicates a significant difference between different groups (*p* < 0.05).

Based on the results of enzyme activity and histopathological evaluations, the CON and S2 groups were selected for intestinal microbiota analysis. After quality filtering and chimera removal, high-quality sequences were clustered into operational taxonomic units (OTUs) at a 97% similarity, providing a robust basis for downstream analysis. An UpSet plot (Figure 9A) was used to visualize the distribution and intersection of OTUs. A total of 120 OTUs were identified across both groups, with 58 OTUs unique to the CON group and 65 OTUs unique to the S2 group. Alpha diversity analysis (Figure 9B) showed that the coverage index for each group exceeded 0.99, indicating adequate sequencing depth. No significant difference was observed in the coverage index between the groups (*p* > 0.05). However, the Shannon, Simpson, and Pielou indices were significantly lower in the S2 group compared to the CON group (*p* < 0.05), indicating a decrease in community diversity and evenness following high-dose Se exposure. In contrast, the Chao1 and ACE indices did not differ significantly between the groups (*p* > 0.05), suggesting that community richness remained relatively stable.

#### 3.8.2. Composition of the Intestinal Microbiota

**Figure 10:**
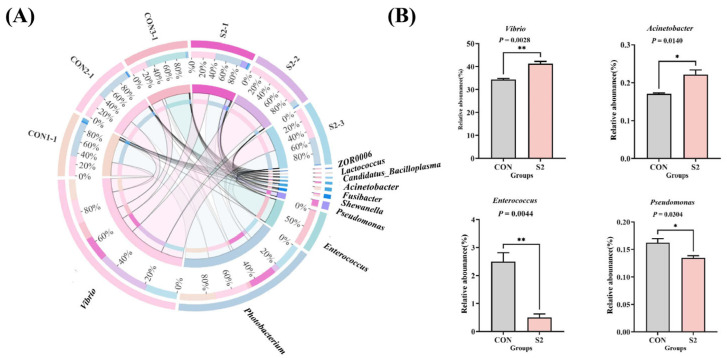
Composition of intestinal microecology of *L. vannamei* by genus. (**A**) Genus-level Circos diagram; (**B**) changes in dominant bacterial genera. * indicates a significant difference between different groups (*p* < 0.05); ** indicates a significant difference between different groups (*p* < 0.01).

Analysis of microbial composition at the genus level revealed that *Vibrio*, *Photobacterium*, *Enterococcus*, *Pseudomonas*, *Shewanella*, *Fusobacterium*, *Acinetobacter*, ZOR0006, *Candidatus Bacilloplasma*, and *Lactococcus* were the dominant genera in both the CON and S2 groups (Figure 10A). Among these, *Vibrio*, *Photobacterium*, and *Enterococcus* were the three most abundant. In the S2 group, the relative abundance of *Vibrio* and *Acinetobacter* significantly increased compared to the CON group (*p* < 0.05), while the abundance of *Enterococcus* and *Pseudomonas* significantly decreased (*p* < 0.05, Figure 10B). These results indicate that acute Se exposure markedly altered the composition of dominant bacterial genera.

#### 3.8.3. Analysis of Intestinal Microbiota Differences

**Figure 11:**
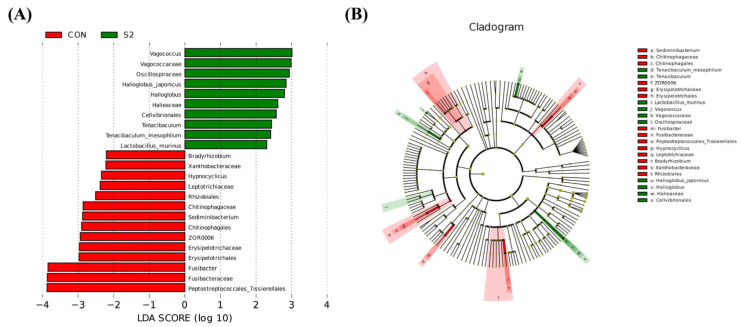
LEfSe difference analysis. (**A**) LEfSe branch plot comparing the CON and Se groups. (**B**) LDA scores of LEfSe-PICRUSt comparison between the CON and Se groups.

To further investigate microbial taxa that differed significantly between groups, linear discriminant analysis effect size (LEfSe) was employed (Figure 11). LDA scores identified 24 taxa with significant differences in relative abundance between the CON and S2 groups. Specifically, the CON group was enriched in 14 taxa, including four orders (Rhizobiales, Erysipelotrichales, Chitinophagales, Peptostreptococcales-Tissierellales), five families (Bradyrhizobiaceae, Chitinophagaceae, Xanthobacteraceae, Erysipelotrichaceae, Fusibacteraceae), and five genera (*Bradyrhizobium*, *Sediminibacterium*, *Hypnocyclicus*, *Fusibacter*, *ZOR0006*). In contrast, the S2 group was enriched in 10 taxa, including one order (Cellvibrionales), three families (Vagococcaceae, Halieaceae, Oscillospiraceae), three genera (*Vagococcus*, *Tenacibaculum*, *Halioglobus*), and three species (*Halioglobus japonicus*, *Tenacibaculum mesophilum*, *Lactobacillus murinus*). These findings suggest that acute Se exposure selectively altered key microbial populations in the intestine.

#### 3.8.4. Analysis of Intestinal Microbial Function

**Figure 12:**
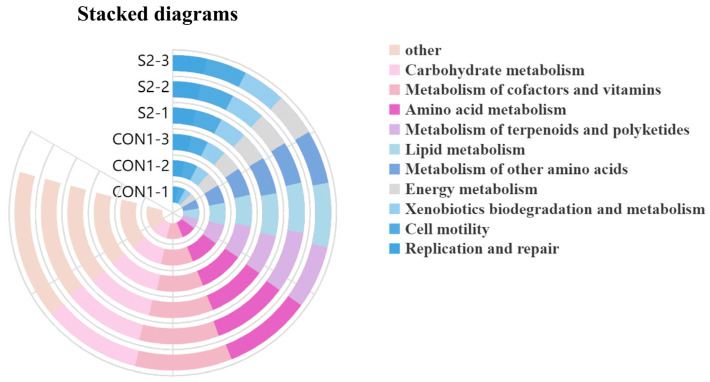
PICRUSt2 functional prediction of intestinal microbiota in *L. vannamei*.

Functional profiling of intestinal microbiota using PICRUSt2 and KEGG database annotations revealed no statistically significant differences in predicted microbial functions between the CON and S2 groups (*p* > 0.05, Figure 12). The top 20 predicted metabolic pathways were conserved across both groups. However, a trend of functional decline was observed in the S2 group, particularly in pathways associated with carbohydrate metabolism (12.80%), lipid metabolism (8.10%), and amino acid metabolism (11.52%). These results suggest that although the overall functional composition remained unchanged, acute Se exposure may suppress the metabolic potential of the intestinal microbiota in *L. vannamei*.

## 4. Discussion

Environmental pollution imposes substantial economic losses on shrimp aquaculture, with heavy metals posing particular risks due to their bioaccumulative toxicity on shrimp growth and survival [22]. Se is a metalloid with transitional properties between metals and nonmetals, and it induces oxidative stress, developmental retardation, elevated mortality, and tissue damage in aquatic organisms when excessively accumulated [23]. Current research has focused on the mechanisms of Se toxicity in fish and waterfowl, while data on dissolved Se toxicity in decapod crustaceans are limited [24,25]. This study employed sodium selenite to simulate aquatic Se contamination, systematically investigating its impacts on survival rate, hepatopancreatic histopathology, oxidative–antioxidant balance, non-specific immune responses, apoptosis, intestinal microbiota composition, and hemocyte transcriptome regulation in *L. vannamei*.

Previous studies have demonstrated that excessive exposure to Se induces toxicological manifestations in aquatic species, including respiratory impairment and energy reserve depletion. Acute Se exposure has been documented to cause 96 h median lethal concentrations (96 h LC_50_) of 1.475 mg L for *Catla catla*, 1.542 mg/ L for *Labeo rohita*, and 1.542 mg /L for *Cirrhinus mrigala fry*, highlighting species-specific susceptibility patterns among Indian major carps [26]. According to certain studies, *L. vannamei* larvae exposed to Se have a 96 h LC_50_ of 3.36 mg/L [27]. In this experiment, the LC_50_ values at 24, 48, 72, and 96 h of Se exposure were 7.12, 3.74, 2.69, and 2.69 mg/L, respectively. These differences in the LC_50_ values may be attributed to the different life stages of the test animals and other abiotic factors. These findings suggest that Se exposure in water leads to a decline in the physiological functions of shrimp, with high Se concentrations posing a significant risk to the survival of *L. vannamei*.

The hepatopancreas of shrimp is a sensitive organ that is highly susceptible to damage from water pollutants [28]. The hepatopancreas of *L. vannamei* consists mainly of branched tubules lined with four different types of epithelial cells (E cells, R cells, F cells, and B cells). Among them, B cells are mainly located at the distal end of the renal tubule and are the largest cells, containing a large vacuole and a basal nucleus, which have the function of secretion [29]. R cells are involved in nutrient storage and detoxification processes [30]. Regarding the effects of Se exposure on liver tissue, previous studies have reported liver cell damage in mice following subacute exposure to selenite diets [31]. Selenite derivatives (such as sodium selenite, Se, and nano-Se) have been shown to cause significant morphological changes in HaCaT cells, affecting their size and shape [32]. In this experiment, exposure to Se at both concentrations led to enlargement of the lobular cavities and cellular necrosis in the hepatopancreas of *L. vannamei*. These studies have shown that high concentrations of Se may damage liver tissue and cause cell necrosis.

Apoptosis is a process of severe cellular damage caused by oxidative stress, which in turn causes extensive damage [33]. Investigations have shown that excessive exposure to Se causes a redox shift toward a more oxidative cellular environment, which in turn triggers apoptosis by directly oxidizing protein thiol groups and indirectly producing ROS [34]. One essential effector enzyme in programmed cell death is caspase-3, the apoptotic executioner protease [35]. In order to preserve organismal homeostasis, it controls the apoptotic removal of damaged cells [36]. The anti-apoptotic protein Bcl-2 promotes cellular survival across biological systems, and its transcriptional downregulation has been demonstrated to correlate with caspase-3/9 activation. Reduced Bcl-2 activity serves as confirmatory evidence of apoptotic progression [37]. There are plenty of mechanistic similarities across taxa: after being stressed to copper, *L. vannamei* has been shown to express caspase-3 in greater quantities [38]. Similarly, following 96 h of exposure, largemouth bass (*Micropterus salmoides*) subjected to Pb showed hepatocyte apoptosis along with Bcl-2 transcriptional downregulation but no changes to Bax expression [39]. These findings are consistent with experimental observations in which increased caspase-3 enzyme activity and decreased Bcl-2 activity, demonstrating inverse regulation between pro-apoptotic caspase-3 and Bcl-2 markers, were detected in the hemolymph of *L. vannamei*, collectively suggesting a Se-induced apoptotic response.

The equilibrium between pro-oxidant factors (endogenous and exogenous environmental contaminants) and antioxidant defenses (enzymatic and non-enzymatic) in organic systems serves as a critical indicator for evaluating the toxic effects of stress-inducing environmental conditions, particularly oxidative damage induced by diverse chemical pollutants [40]. Oxidative stress arises when ROS overwhelms cellular defenses, leading to macromolecular damage in proteins, cellular membranes, and DNA [41]. Se toxicity is attributed to oxygen radical generation, which induces DNA damage, lipid oxidization, and premature protein degradation [42]. The results showed that Se exposure weakened the antioxidant defense of *L. vannamei*, produced excessive reactive oxygen species, and caused oxidative damage. MDA, a terminal product of lipid peroxidation, usually indicates the severity of oxidative damage [43]. Se-induced oxidative stress in other models correlates with elevated MDA levels and glutathione redox shifts [44]. In this study, the activities of ROS enzyme increased during acute Se exposure, while MDA levels showed no significant change, indicating that *L. vannamei* may be in the stage of oxidative damage [45]. Antioxidant enzymes, including SOD, CAT, PO, and GSH-PX, play pivotal roles in organismal defense mechanisms [46]. SOD scavenges superoxide radicals by catalyzing their conversion to hydrogen peroxide (H_2_O_2_), while GSH-PX eliminates H_2_O_2_ and lipid hydroperoxides, mitigating oxidative injury [47]. PO, a vital part of invertebrate innate immunity, uses melanization pathways to mediate phagocytic activity and cellular defense activation [48]. These enzymes exhibit synergistic activity in counteracting lipid peroxidation and free radical propagation. Notably, in Atlantic salmon (*Salmo salar*), elevated Se levels induce oxidative stress and perturb lipid metabolism [49]. Similar dose-dependent biphasic responses in SOD and CAT activities—initial activation followed by suppression—have been documented in clams exposed to cadmium ions (Cd^2+^) [50], while crabs demonstrate enhanced chromium (Cr^6+^) tolerance via SOD-mediated hydroxyl radical scavenging [51]. Results indicated that Se exposure markedly increased SOD, PO, and GSH-PX activities in *L. vannamei* hemolymph, indicating activated cellular antioxidant defenses; however, CAT activity did not change, possibly because GSH-PX preferentially cleared H_2_O_2_ in this system [52]. In summary, it is speculated that Se exposure induces ROS production, causes oxidative stress, and leads to oxidative damage and apoptosis.

When evaluating the toxicological effects of metal exposure, crustacean immunoregulatory responses serve as vital indicators [53]. LYS is an important natural immune factor involved in the body’s immune response to pathogenic bacteria and various stressors [54]. ACP and AKP belong to the group of phosphohydrolases, which are important marker enzymes for lysozyme to break down invading bacteria and viruses, and shrimp respond to environmental stressors by increasing ACP and AKP activity to boost immunity [55]. Previous data have shown that low concentrations of environmental pollutants promote stimulus responses in organisms, while inhibition and toxicity occur under the influence of high doses of environmental pollutants [56,57]. This conclusion is also confirmed by the results of Pb and Cr stress on aquaponics [58]. Similarly, the results of this study also showed that the activity of hemocyte LYS, ACP, and AKP increased under Se exposure, indicating that Se exposure stimulates the production and release of immune-related enzymes, activating the immune system to eliminate cells damaged by exposure.

Growing evidence indicates that heavy metal stress elicits transcriptomic alterations associated with significant perturbations in biological pathways, including oxidative stress, immune responses, metabolism, and apoptosis [59]. Previous studies suggest that Se-induced dysregulation of target molecules through thiol modifications may disrupt signaling pathways governing cellular survival and apoptosis [60]. Transcriptomic profiling in this study further elucidated shrimp response mechanisms to Se via GO and KEGG enrichment analyses. GO and KEGG enrichment showed that DEGs were mainly involved in signal transduction, ribosome biogenesis, and cell cycle regulation, indicating that Se disrupts cellular homeostasis by interfering with protein synthesis and regulatory pathways. In particular, the process of ribosomal biogenesis is a central rate-limiting process for cellular growth and proliferation, which entails the transcription, processing, and assembly of precursor rRNA (pre-rRNA) with ribosomal proteins [61]. Inhibition of the NOP56 gene may interfere with the maturation of the pre-rRNA and even the stability of the RNA secondary structure, thereby disrupting ribosome formation and reducing the efficiency of ribosomal translation [62]. Studies show conserved ribosomal pathway responses to metal exposure, such as upregulation of ribosomal protein genes in cadmium-exposed zebrafish (*Danio rerio*) liver tissues [63]. This compensatory upregulation under ribosomal synthesis inhibition suggests a negative feedback mechanism, which is consistent with experimental observations of elevated expression of ribosome biogenesis-related genes (*Nop60B*, *NOP58*, *NOP56*, *Utp14a*, *PWP2*) under Se exposure [64]. Under contamination stress conditions, cells enhance rRNA stability and ribosomal function by upregulating rRNA-modification-related genes, especially methylation and pseudouridine [65]. Notably, an inverse relationship between immune defense and ribosomal gene activation caused by Se may compromise immunocompetence through resource reallocation.

The lysosomal system, a dynamic organelle network, mediates nutrient assimilation, metabolic regulation, macromolecular degradation, and signal transduction [66]. In addition, lysosomal membrane integrity has been used to indicate the effects of contaminants on invertebrates [67]. Reduced *MANBA* expression induces lysosomal structural and functional alterations, blocking endocytosis and autophagy, ultimately triggering inflammatory cell death and fibrosis following toxic insult [68]. Downregulation of *GBA*, which facilitates lysosomal macromolecule digestion, indicates Se-induced lysosomal dysfunction [69]. Heavy metal exposure typically induces lysosomal membrane destabilization and associated enzymatic dysregulation, as evidenced in this study by suppressed lysosomal enzyme genes (*LIPF*, *PLA2G15*, *GBA*, *LCP2*, *MANBA*) following Se exposure. These findings collectively demonstrate that Se exposure induces lysosomal membrane destabilization in *L. vannamei,* impairing critical cellular clearance mechanisms.

The eukaryotic cell cycle enables organismal growth and tissue repair through regulated cellular proliferation [70]. Previous studies have shown that the highly cytotoxic exogenous substance cypermethrin, after exposure, leads to widespread cell cycle disruption by significantly promoting the expression of *CDK 2*, *CDK 4*, *CDK 7*, *CDK 8*, *CDKN 2A*, and *RB 1* [71]. Cyclin-dependent kinases (CDKs), central regulators of cell cycle progression, also modulate apoptotic pathways [72]. It has been proposed that ATR controls DDR signaling and repair pathways, and also regulates cell cycle checkpoint control and cell death (apoptosis) in response to genotoxic stress. DNA damage activation is found in ATR in tumors and cancer cells, which in turn induces *WEE 1* expression, resulting in cell cycle arrest in the G2/M phase [73]. The experimental results demonstrated Se-induced upregulation of cell cycle-related genes (*Wee1*, *Atr*, *CDK7*, *ccnb2*, *ORC4*), suggesting compensatory activation of cell cycle checkpoints to mitigate Se-triggered DNA damage and apoptosis in *L. vannamei.*

In shrimp, the pancreatic secretory route regulates the enzymatic regulation of glucose metabolism, fatty acid β-oxidation, and amino acid digestion and absorption [71]. Previous reports have suggested that excess metals, such as Cr and Zn, can lead to pancreatic dysfunction [74]. Cr exposure activated the secretion of Mantis Shrimp glandular protein, and the pancreatic secretion pathway up-regulated *GNAS* and *ATP1B* [75]. In this study, the *Cpa2*, *SLO*, and *Rho1* genes associated with the pancreatic secretory pathway were upregulated, indicating that Se exposure also affected pancreatic secretion. The chloride channel appendage *CLCA* gene is involved in a variety of biological processes, including cell differentiation, adhesion, apoptosis, and airway inflammation, with *CLCA 2* responsible for mediating tumor cell invasion and the absence of *CLCA2* promoting the transformation of epithelial cells into mesenchymal cells, suggesting a higher chance of cancer metastasis [76]. *CLCA4* may inhibit epithelial–mesenchymal transition (EMT) through PI3K/ATK signaling, thereby inhibiting cell migration and invasion [77]. In this experiment, Se downregulated the expression of *CLCA4* and *CLCA2* genes, indicating that Se increased the risk of bacterial invasion of *L. vannamei*.

Microbial communities play a crucial role in regulating the homeostasis of host physiological functions and resistance to environmental pollutants [78]. Heavy metals have been demonstrated to pose significant threats to intestinal microbiota, leading to alterations in host health status [79,80] The toxic effects of Se are closely associated with its intestinal absorption dynamics [81]. The present study demonstrated that, similar to Cd-induced alterations in the intestinal microbiota of Scylla paramamosain [82] and Pb-driven disruption of microbial balance in *Procambarus clarkia* [83], high-concentration Se exposure resulted in an increase in unique OTU counts in the intestinal microbiota of *L. vannamei*. Alpha-diversity indices, reflective of host intestinal health [84]], exhibited significant reductions in Shannon, Simpson, and Pielou indices in Se-stressed groups, indicating compromised intestinal ecosystem stability. At the genus level, Se exposure induced a pathogenic shift characterized by increased relative abundances of *Vibrio* and *Acinetobacter*, alongside decreased proportions of beneficial *Enterococci* and *Pseudomonas*. *Vibrio*, recognized as a major aquaculture pathogen, causes substantial economic losses through vibriosis outbreaks in crustacean farming systems [85]. Likewise, *Acinetobacter* is linked to bacterial septicemia and shrimp mortality [86]. The increase of *Acinetobacter* and *Vibrio* in this experiment indicates the occurrence of intestinal dysfunction in shrimp under Se exposure. The decrease in the abundance of probiotic Enterococci—which are known to improve feed utilization, immune function, and antioxidant capacity in aquatic species [87]—and *Pseudomonas*, which stimulates intestinal epithelial proliferation via quorum-sensing mechanisms [88,89], indicates that Se will reduce the beneficial bacteria of shrimp. PICRUSt2 functional prediction also showed reduced metabolic pathway signals (carbohydrate, lipid, and amino acid metabolism) under Se exposure, which is consistent with observed microbiota dysbiosis. All these findings together show that Se exposure upsets the microbial balance, compromising intestinal barrier integrity and metabolic homeostasis in *L. vannamei*. The concordance between microbial community shifts and transcriptomic alterations in ribosomal/pancreatic pathways highlights the systemic nature of Se toxicity, where microbiota dysbiosis exacerbates host metabolic and immunological dysfunction.

## 5. Conclusions

This study demonstrates that excessive Se exposure disrupts multiple physiological systems in *L. vannamei*, inducing hepatopancreatic damage, oxidative stress, apoptosis, and immune dysregulation. Transcriptomic analysis revealed alterations in pathways related to ribosome biogenesis, lysosomal function, and the cell cycle. Additionally, aquatic Se stress adversely affected intestinal health in *L. vannamei.* Overall, these findings provide critical insights for ecological risk assessment and safety threshold determination regarding aquatic Se exposure in commercial shrimp farming systems.

## Figures and Tables

**Table 1:** Sequences of the primers used for qPCR.

Primer	Sequences (5′-3′)	Sequences (5′-3′)
ncbi_113823493	F: TCGAGAGTGGTTGTGCAGAC	R: GTCGTTGTTCGGGTGTAGGT
ncbi_113819543	F: ACCTTCGAGAGTGGTTGTGC	R: TGAAGTGTTCGACGGAGACG
ncbi_113830564	F: TGTGAAGACGGTCTGAAGCC	R: ACACATCCAGTGTCTGCGAG
ncbi_113821441	F: TCTTTAGCTGCTCTGCCACC	R: TGCAGCCACCCATACTGAAG
ncbi_113805388	F: ATGTCCAGCTCGGGCTAATG	R: AGCCACTAACAGGGTCAAGC
ncbi_113825012	F: TCTAACGAGAACTACAGCGAGTGG	R: CGGTCTTGATGGTGACGACATTG
ncbi_113814977	F: AGGCTGGAGCTGATCCTAACATC	R: GCCGTGTCGCCATCATTATCG
ncbi_113821612	F: CCGACGCCGACCCTTGG	R: CGTAGCCTCCTCTTCCGTAGTAG
ncbi_113809493	F: GAAGGAAAGCGACACTCACACTG	R: CGTAGCGGAGGCGAAGGAC
ncbi_113802274	F: GTGATGTGCTGTGGATGTGACTC	R: CTGACTGGTGATCTGCTTCTTGAC
LvEF-1α	F: TGCACCACGAAGCCCTTAC	R: CAGGGTGGTTGAGGACGATC

**Table 2:** Cumulative mortality rate (%).

Exposure Time (h)	Se Concentration (mg/L)						LC_50_ (mg/L)
	0.1	0.5	2.5	12.5	62.5	125	
24	0	0	5	80	100	100	7.12 (5.16–9.81)
48	0	5	20	100	100	100	3.74 (2.71–5.15)
72	5	10	40	100	100	100	2.69 (1.74–4.16)
96	5	10	40	100	100	100	2.69 (1.74–4.16)

Note: Cumulative mortality (%) and the intermediate lethal concentration (LC_50_, 95% confidence limit) of *L. vannamei* at different Se concentrations.

**Table 3:** Statistics of the sequencing data.

Samples	Raw Data (bp)	Clean Data (bp)	Q20 (%)	Q30 (%)	GC (%)
CON1-1	6500717400	6417446174	97.20%	92.74%	47.67%
CON1-2	6030712500	5956039994	96.87%	92.02%	46.36%
CON1-3	7480366500	7376684029	97.18%	92.66%	45.94%
S2-1	6423535500	6351128194	97.07%	92.36%	45.90%
S2-2	6968606700	6884621547	97.18%	92.58%	46.78%
S2-3	7259163600	7172233369	97.09%	92.42%	46.11%

Note: Sample: sample name; Raw Data: the number of reads in the raw data; Clean Data: the number of high-quality reads filtered from the original data; Q20: the percentage of the base number with a Qphred value of not less than 20 to the total base number; Q30: the percentage of the base number with a Qphred value of not less than 30 to the total base number; GC: GC content in high-quality reads.

**Table 4:** Effect of selenium exposure on KEGG pathways related to *L. vannamei*.

Gene ID	Log_2_(fc)	Symbol	Description
*Lysosome*			
ncbi_113801959	−4.42	*LIPS*	triacylglycerol lipase
ncbi_113805268	−1.11	*PLA2G15*	PREDICTED: group XV phospholipase A2-like
ncbi_113806002	−1.31	*GBA*	PREDICTED: glucosylceramidase-like isoform X2
ncbi_113808797	−1.88	*LCP2*	cathepsin l
ncbi_113809383	−1.31	*MANBA*	PREDICTED: beta-mannosidase-like
*Ribosome biogenesis in eukaryo*			
ncbi_113800232	1.33	*Nop60B*	PREDICTED: H/ACA ribonucleoprotein complex subunit 4
ncbi_113802117	1.26	*NOP58*	Nucleolar protein NOP5
ncbi_113803266	1.41	*NOP56*	Nucleolar kke/d repeat protein
ncbi_113804709	1.98	*Utp14a*	PREDICTED: U3 small nucleolar RNA-associated protein 14 homolog A-like
ncbi_113805050	1.48	*PWP2*	PREDICTED: periodic tryptophan protein 2 homolog
*Pancreatic secretion*			
ncbi_113802097	−1.49	*Cpa2*	carboxypeptidase B, partial
ncbi_113805576	3.47	*CLCA4*	Calcium-activated chloride channel regulator 4, 30 kDa form
ncbi_113807409	−1.02	*Oslo*	calcium-activated potassium channel transcript variant 1
ncbi_113809844	−5.80	*Rho1*	RhoA
ncbi_113813375	3.40	*CLCA2*	Calcium-activated chloride channel regulator 4, 30 kDa form
*Cell cycle*			
ncbi_113822117	1.77	*Wee1*	PREDICTED: wee1-like protein kinase
ncbi_113822118	1.30	*Atr*	PREDICTED: serine/threonine-protein kinase ATR-like isoform X2
ncbi_113808550	1.28	*CDK7*	cyclin-dependent kinase 7
ncbi_113821379	1.99	*ccnb2*	cyclin B
ncbi_113828355	1.68	*ORC4*	PREDICTED: origin recognition complex subunit 4
*Pyrimidine metabolism*			
ncbi_113800828	1.21	*POLR3A*	PREDICTED: DNA-directed RNA polymerase III subunit RPC1 isoform X2
ncbi_113800946	1.62	*pola1*	PREDICTED: DNA polymerase alpha catalytic subunit-like
ncbi_113801001	1.35	*Pole*	PREDICTED: DNA polymerase epsilon catalytic subunit A-like
ncbi_113802524	1.38	*Polr1b*	DNA-directed RNA polymerase I subunit RPA2
ncbi_113802842	1.60	*polr1e*	PREDICTED: DNA-directed RNA polymerase I subunit RPA49-like

## Data Availability

The data that support the findings of this study are available upon request from the corresponding author. The data are not publicly available due to privacy or ethical restrictions.
